# Impact of Salting Techniques on the Physio-Chemical Characteristics, Sensory Properties, and Volatile Organic Compounds of Ras Cheese

**DOI:** 10.3390/foods12091855

**Published:** 2023-04-29

**Authors:** Dina A. Amer, Abdinn A. M. Albadri, Hanaa A. El-Hamshary, Yasser Nehela, Mohamed Y. El-Hawary, Abeer H. Makhlouf, Sameh A. Awad

**Affiliations:** 1 Department of Food Science and Technology, Faculty of Agriculture, Tanta University, Tanta 31527, Egypt; 2 Department of Biology, College of Science, King Khalid University, Abha 62529, Saudi Arabia; 3 Department of Agricultural Botany, Faculty of Agriculture, Tanta University, Tanta 31527, Egypt; 4 Department of Plant Pathology, Citrus Research and Education Center, University of Florida, Lake Alfred, FL 33850, USA; 5 Department of Agricultural Botany, Faculty of Agriculture, Minufiya University, Shibin El-Kom 32511, Egypt; 6 Dairy Microorganisms and Cheese Research Laboratory (DMCR), Department of Dairy Science and Technology, Faculty of Agriculture, Alexandria University, Alexandria 21545, Egypt; sameh.awad@alexu.edu.eg

**Keywords:** Ras cheese, salting, ripening, volatile compounds, GC-MS, milk, dairy

## Abstract

Ras cheese is the main Egyptian hard cheese that is well-known worldwide. Herein, we investigated how different salting techniques affect the physio-chemical properties, sensory properties, and volatile compounds of Ras cheese over a six-month ripening period. Five Ras cheese treatments were made from pasteurized cow’s milk using various salting techniques: traditional salting of Ras cheese, salting by applying all of the salt to the curd after the entire whey drainage, salting by applying all of the salt to the curd after half to two-thirds of the whey drainage, salting in a brine solution for 24 h without dry salting, and salting in a brine solution for 12 h and then dry salting. The obtained results by GC-MS recorded that thirty-eight volatile compounds were identified in Ras cheese treatments after six months of ripening, and the development of volatile compounds was affected by the salting technique as well as the ripening period of the cheeses, which played a major role in the type and concentration of volatile compounds. Results revealed that there are six esters, 15 fatty acids, five ketones, two aldehydes, four alcohols, and eight other compounds identified in most treatments. Some physio-chemical characteristics and sensory properties were found to have high correlations with the storage period, while some others have low correlations during the ripening period.

## 1. Introduction

Cheese is a significant part of the food consumed in numerous countries. Ras cheese is the main Egyptian hard cheese that has become the most well-known and delicious hard cheese in Egypt and the Arab world [1]. Ras cheese is one of the most famous hard cheeses in Egypt. It is usually made from raw cow’s milk or a mixture of cow and buffalo milk under artisanal conditions without the use of specific starters, and it is consumed when it has a strong, sharp flavor after 3 to 6 months. The ripening period of Ras cheese makes it a perfect product for consumption. The Egyptian Organization for Standardization and Quality emphasizes the implementation of quality standards which indicate that all types of cheese should be made from pasteurized milk, which affects the characteristics and properties of cheese throughout ripening [2,3]. Ras cheese is ripened at temperatures ranging from 9 °C to 12 °C [4].

Ras cheese is valued for its portability as well as the elevated content of compounds such as fat, protein, calcium, and phosphorus. Ras Cheese has longer shelf life compared with milk and other dairy products. Different aspects of Ras cheese were determined by El-Leboudy et al. 2014 as the assessment of sanitary measures of Ras cheese was undertaken through microbiological evaluation of cheese and its processing environments in the traditional manufacturing dairy industry [5]. Additionally, the physio-chemical properties, sensory properties, and textural characteristics of Egyptian Ras cheese throughout the ripening period were previously studied by El-Leboudy et al. 2014, Gunasekaran 2002 and Patrick et al. 2004 [3,5,6,7]. Salt is a crucial ingredient in cheese because it has a role in improving the safety, quality, and flavor of the cheese. Salt also enhances the texture of the cheese through the hydration state into a viscous texture and regulates the activity of microorganisms [8,9]. Various salting techniques influence the functional properties of natural cheese. For instance, the method of salting influences the mineral content of cheese more than it affects the textural properties. Dry-salted cheese could be considered of greater dietary value than industrially manufactured brine-salted. In addition, dry-salted cheeses exhibited significantly lower hardness and fracturability values compared to cheeses that were brine-salted [10,11].

Numerous people prefer a diet without salt, so they would choose dairy products that with reduced salt content over hard cheese with high levels of sodium. The technique of lowering the sodium content of salt in cheese is important in the production of cheese that satisfies consumer requirements. The content of salt in cheese is essential for advancing strategies to produce low sodium cheese that satisfies consumer requirements. Decreasing the salt content in cheese involves the enrichment of whey separation and adjusting the moisture content of the final product. Furthermore, this is attributed to the domination, viability, and metabolism of starter culture, and secondary microorganisms in cheese which might raise the volatile compounds throughout the ripening period that control the texture of final products [12].

Salt is an important provider influencing the aroma of cheese. Moreover, salt might increase the concentration of flavor during the ripening period and reduce the aroma of bitterness in cheese. The formation of bitterness in cheese might be a result of salt and the activity of starter culture during the ripening period [13,14]. The flavor of the cheese is the most relevant criterion that indents consumer choice and acceptance. It is the result of the adjusted balance and concentration of a wide variety of volatile flavor compounds during ripening, released by enzyme reactions rather than by chemical interactions. The flavor of matured cheese is influenced by proteolysis and lipolysis [15]. Many factors in cheese, such as milk enzymes, rennet, and starter culture, cooperate in the degradation of milk composition: fat, protein, and lactose. Differences in derived compounds depend on cheese variety, procedure, and ripening requirements [16].

Cheese is an energetic biochemical product, because of a series of sequential changes that take place during the cheese manufacturing process, and it also undergoes considerable changes during ripening. The enrichment of cheese quality, as well as the modulation of cheese flavor, are targets of great interest not only for industry but also for research areas. The flavor profiles of cheeses are complex, varied, and type-specific [7,17]. Hard cheese has different volatile components that are associated with many chemical groups such as acids, esters, aldehydes, ketones, alcohols, and sulfur compounds that are well-known in different cheeses [18,19]. These compounds are accountable for the taste and flavor of hard cheese [20].

Ripening includes different pathways such as glycolysis, lipolysis, and proteolysis that occur in cheese during ripening which result in the flavor, texture, and appearance [21]. Throughout the ripening of cheese, biochemical changes occur in cheese compounds, including the metabolism of lactose and citrate, lipolysis, and proteolysis, while secondary biochemical events contribute to many volatile flavor compounds and contain the metabolism of fatty acids and amino acids [22]. The microbiological changes of cheese through the ripening period involve the lysis of starter microorganisms as well as the growth of non-starter lactic acid bacteria [23,24].

Sensory properties affected by flavor and texture play an important role in consumer acceptance. The flavor, color, and texture are the main criteria that determined the acceptability of ripened cheese. During the ripening period, cheese obtains its properties through many chemicals, and microbiological and biochemical changes whereby protein, fat, and reaming lactose are broken down into primary products which are remotely degraded into secondary products [25]. Comprehensive sensory assessment of cheese has often been chosen for texture measurements. Several experiments have been developed to correlate sensory with texture characteristics such as the assessment of proteolysis, water-soluble nitrogen, electrophoresis, free amino groups, lipolysis, and texture profile analysis for hard cheese [26]. For this purpose, several instruments were used such as the “Texture Analyser” which is well-known for measuring the hardness, cohesiveness, springiness, and chewiness of hard cheese and how they affect the sensory properties of cheese during ripening. Moreover, they attempted to correlate sensorial with physio-chemical properties of hard cheese in Egypt as Ras cheese, which is made from raw cow’s milk or a mixture of cow’s and buffalo’s milk [2,3,27].

The objective of this study was to elucidate the effect of different salting techniques on the physio-chemical characteristics and volatile compounds of Ras cheese. Therefore, the present work focuses on an evaluation of the chemical characteristics’ sensory properties as well as the identification of the flavor compounds of experimental Ras cheese during a 6-month ripening period.

## 2. Materials and Methods

### 2.1. Materials

Fresh raw full-fat cow’s milk was obtained from the dairy farm of the Faculty of Agriculture, Alexandria University. For starter cultures, freeze-dried lactic culture (YF-L904) for direct vat set (DVS) consisting of *Streptococcus thermophilus* and *Lactobacillus delbreuckii* subsp. *bulgaricus* was obtained from Chr. Hansen Lab., (Hoersholm, Denmark). The commercial rennet (CHY-MAX Powder Extra NB, 3.17 × 105Su (Soxhlet units)/g) used was obtained from Chr. Hansen, Inc. (Hoersholm, Denmark). Calcium chloride, sodium chloride, and paraffin wax were obtained from the EL-Nasr Company, Alexandria, Egypt. The ingredients and chemical reagents used were of analytical grade.

### 2.2. Ras Cheese Manufacture

Ras cheese was made in the dairy pilot plant of the Department of Dairy Science and Technology, Alexandria University. Raw cow’s milk (3.5% fat) was pasteurized at 63 °C for 30 min and cooled to 38 °C, then the milk was transferred to a cheese vat. The control sample of Ras cheese was made according to Hofi et al., 1970 [27], and the other four treatments of Ras cheese were made according to Awad, 2006 [3] with slight modifications in the salting process as described previously [28]. Commercial starter culture (5 units/100 kg) and CaCl_2_ at level 0.012% (*w*/*w*) were added to the milk. After ripening at 35 °C for 1 h, the milk was clotted by adding microbiological coagulating enzyme for 45 min, then the curd was cut with longitudinal and transverse knives. Scalding was carried out by raising the temperature gradually to 45 °C over 60 min and holding for 15 min. Whey was drained, and the salting of each treatment was performed. The salting of the control sample of Ras cheese was performed traditionally by applying the (5%) salt after about 2/3 whey drainage and then adding dry salting of the cheese wheels. Treatments were: T1, applying the salt (5%) after the drainage of the whole whey, then dry salt was added to the wheels; T2, applying the salt (5%) after about 2/3 of whey drainage; T3, applying the salt in a brine solution (24%) for 24 h; and T4, applying the salt in a brine solution (24%) for 12 h, then dry salt was added to the wheels.

Subsequently, the cheese curd was formed and pressed for 24 h, and then the wheels were turned up for 24 h. Cheese wheels were allowed to ripen under controlled temperature and relative humidity for about 13 ± 2 °C—85% up to 16 months, respectively. Control and experiment samples were analyzed at fresh (2 days of manufacturing), 1, 2, 4, and 6 months for physicochemical and sensory evaluation, as well as volatile flavor compounds.

### 2.3. Physio-Chemical Analysis of Ras Cheese

Cheese samples were analyzed according to the methods of the Association of Official Analytical Chemists [29]. The titratable acidity and pH value of samples were measured in a slurry prepared by macerating 20 g of grated cheese in 20 mL of deionized water using a pH meter (Mi 151 PH, ORP, Temperature Bench Meter) while titratable acidity was measured by recording the number of the N/10 sodium hydroxide solution (0.1 Normal NaOH) that was used to reach the endpoint. Fat was determined using Gerber’s method, and total nitrogen (%) was determined using the Kjeldahl method and the 6.38 factor to convert the nitrogen content to proteins. The ash content was determined using muffle furnaces at 550 °C. The salt content was determined using AgNO_3_. 0.05 N and Potassium Chromate (2%), according to Pearson’s method [30,31].

### 2.4. Sensory Evaluation

The sensory qualities of cheese samples were assessed after 6 months of the ripening period via a regular scoring panel of staff members of the Dairy Science & Technology Department at Alexandria University. The panelists were surveyed for flavor and texture [32]. The scale was as follows: 1, inadequate; 2, enough; 3, acceptable; and 4, very good. The texture was graded on a scale of 1 (soft), 2 (average), 3 (hard), and 4 (extremely hard). The panelists were asked to assign the samples an overall grade of 1–100.

### 2.5. Assay of Volatile Organic Compounds (VOCs)

Volatile compounds of the cheese samples were identified using purge-and–trap thermal desorption cold-trap (TDCT) gas chromatography-mass spectrometry (GC-MS) [20] as follows. Prepared 20 mL of cheese slurry and double-distilled water (1:2 *w*/*v*). The samples were purged with 150 mL min^−1^ helium gas for 30 min at 42 °C and volatile components were trapped on an absorbent trap containing a carbon trap (80 mg, 20–40 mesh, Supelco). The trapped compounds were transferred onto a capillary column of a gas chromatograph, using the Chrompack PT1 injector, by heating the trap oven: Initial temp 50 °C for 4 min, ramp 6 °C/min to 150 °C, hold 2 min, ramp 6 °C/min to 200 °C, hold 0 min, ramp 6 °C/min to 280 °C, hold 2 min, Inj = 280 °C, Volume = 1 µL, Split = 20:1, Carrier Gas = He, Solvent Delay = 5.00 min, Transfer Temp = 280 °C, Source Temp = 200 °C. The conditions for the chromatographic separation and mass spectrometry have been prepared according to Engels et al., [20]. Volatile compound compositions were assigned by spectrum explanation, comparison of the spectra with bibliographic data, and comparison of retention times with those of reference compounds, using (GC-MS) Perkin Elmer/Model, Clarus 580/560 S. Scan: 50 to 620 Da, Column (Elite-5MS, 30 m 0.25 mm, and ID 0.25 um).

### 2.6. Statistical Analysis

The experiment was carried out using a completely randomized design (CRD) with three replications per treatment. All data were statistically analyzed using the analysis of variance (ANOVA) followed by Tukey’s honestly significant difference (HSD) test as a post-hoc analysis to compare between means (*p* < 0.05). The statistical technique of Principle Component Analysis (PCA) was used in analyzing the obtained results [33].

## 3. Results

**Figure 1:**
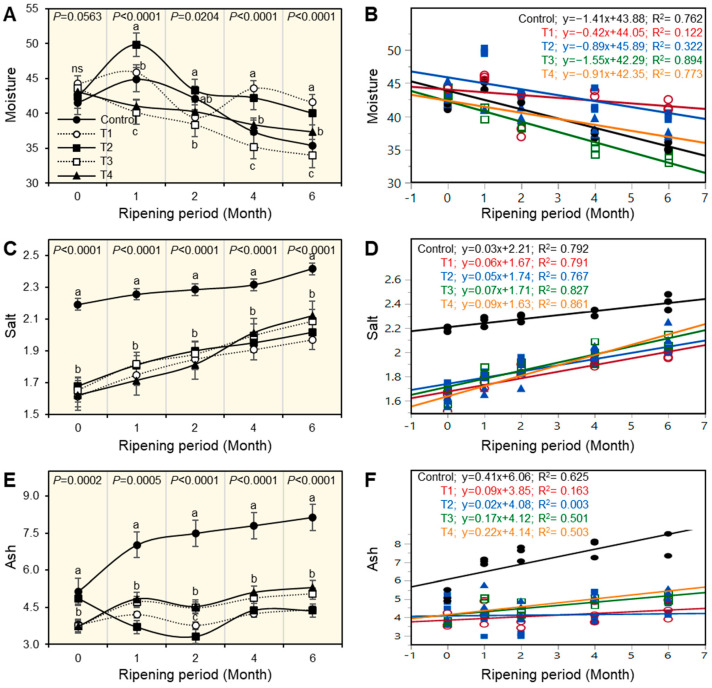
Effect of different salting techniques on physical properties of Ras cheese. (**A**) Moisture content, (**B**) Simple linear regression (SLR) between moisture and ripening periods, (**C**) Salt%, (**D**) SLR between salt% and ripening periods, (**E**) Ash, and (**F**) SLR between ash and ripening periods. Values represent the means ± standard deviation (means ± SD) of three biological replicates (*n* = 3). Different letters indicate statistically significant differences between treatments (*p* < 0.05).

**Figure 2:**
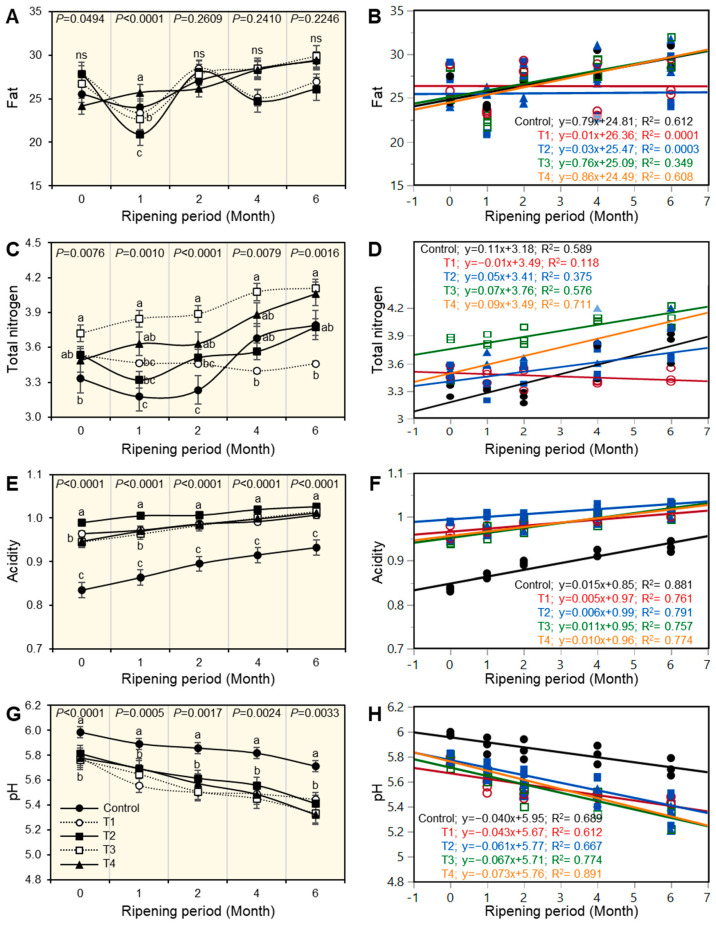
Effect of different salting techniques on chemical and physical properties of Ras cheese. (**A**) Fat, (**B**) Simple linear regression (SLR) between fat and ripening periods, (**C**) total nitrogen, (**D**) SLR between total nitrogen and ripening periods, (**E**) acidity, (**F**) SLR between acidity and ripening periods, (**G**) pH values, and (**H**) SLR between pH values and ripening periods. Values represent the means ± standard deviation (means ± SD) of three biological replicates (*n* = 3). Different letters indicate statistically significant differences between treatments (*p* < 0.05).

**Figure 3:**
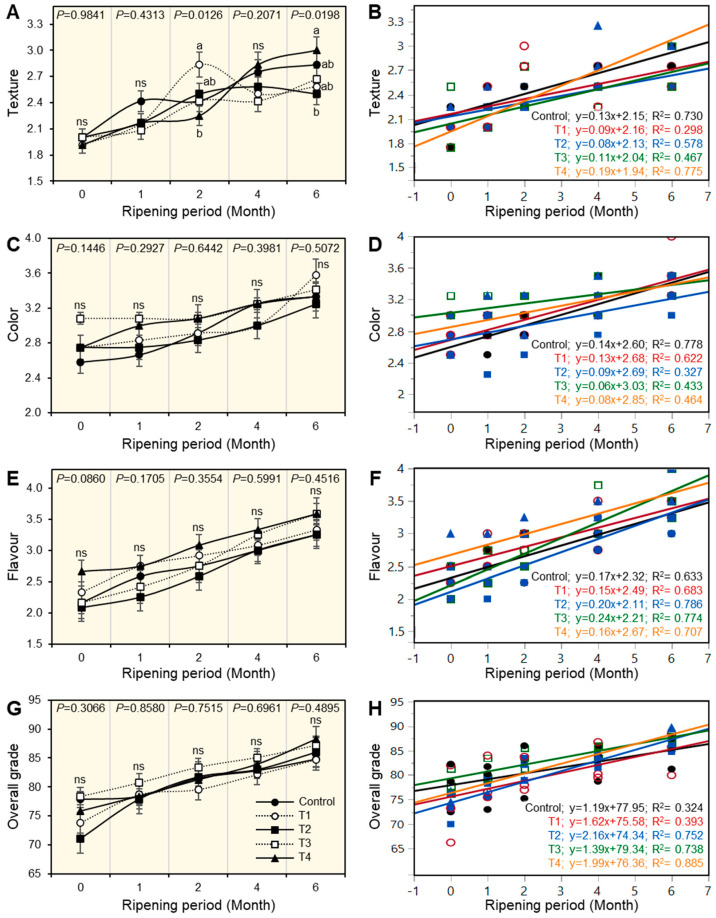
Effect of different salting techniques on sensory properties of Ras cheese. (**A**) Texture, (**B**) Simple linear regression (SLR) between texture and ripening periods, (**C**) color, (**D**) SLR between color and ripening periods, (**E**) flavor, (**F**) SLR between flavor and ripening periods, (**G**) overall grade, (**H**) SLR between overall grade and ripening periods. Values represent the means ± standard deviation (means ± SD) of three biological replicates (*n* = 3). Different letters indicate statistically significant differences between treatments (*p* < 0.05).

**Figure 4:**
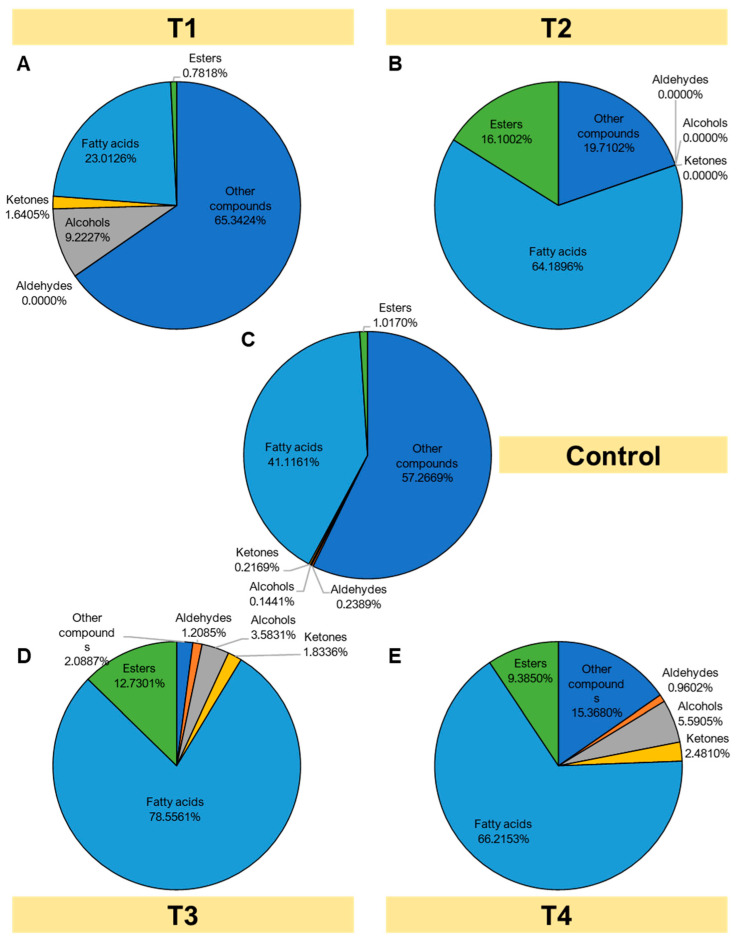
Percentages of volatile organic compounds (VOCs) of Ras cheese treated with different salting techniques over a six-month ripening period. (**A**) T1, (**B**) T2, (**C**) Control, (**D**) T3, and (**E**) T4.

**Figure 5:**
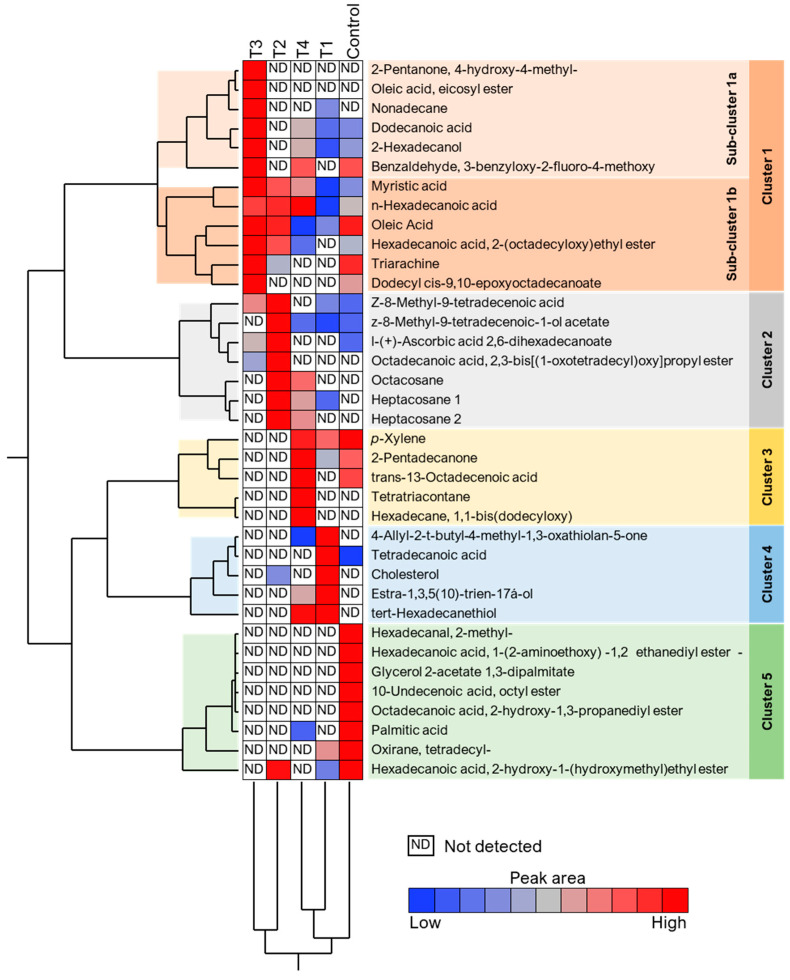
Hierarchical cluster analysis (HCA) and heat map using the abundances of volatile organic compounds (VOCs) of Ras cheese treated with different salting techniques over the six-month ripening period. Rows represent VOCs abundance and columns represent treatments. Cells are colored based on abundance. Red represents high abundance while blue represents low abundance.

**Figure 6:**
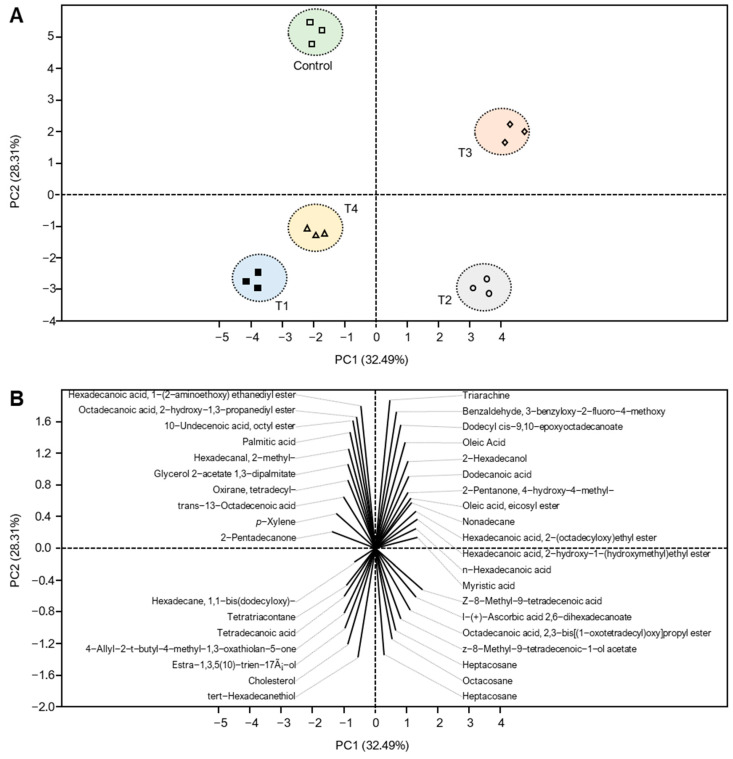
Principal components analysis (PCA) and its associated biplot of volatile organic compounds (VOCs) of Ras cheese treated with different salting techniques over a six-month ripening period. (**A**) PCA-scatterplot using the abundances of all volatiles and its PCA-biplot (**B**).

Changes in the physiochemical properties of experimental Ras cheese are shown in Figure 1, Figure 2, Figure 3, Figure 4, Figure 5 and Figure 6. The basic composition of Ras cheese was determined in samples over 6 months.

### 3.1. The Moisture Content of Ras Cheese

On observing the moisture content of Ras cheese treatments, it could be observed that there were significant differences among all cheese treatments (*p* < 0.05), when fresh and during the ripening period, as affected by salting techniques (Figure 1A). Fresh control cheese had the lowest moisture content, while T1 cheese had the highest content compared with other treatments. The moisture content decreased in all cheese treatments as the ripening period progressed; this trend was much more pronounced in control and T3, which had the lowest moisture content at the end of the ripening period.

The simple linear regression (SLR) as presented in Figure 1B was used to better understand the relationship between moisture content and the ripening period of the cheese. Results illustrated that the relationship between moisture content and ripening period was negatively correlated. The moisture content of all cheeses decreased as the ripening period progressed. Treatments C, T3,and T4 had high negative correlations: C: y = −1.4x + 43.88; T3: y = −1.55x + 42.29; and T4: y = −0.9x = 42.35. However, T1 and T2 had a low negative correlation.

### 3.2. The Salt Content (%) of Ras Cheese

The salt content can indicate accessibility due to helping in adding the flavor of cheese. It also controls the bacteria that grow inside the cheese, which may cause undesirable flavor effects on the acceptability. Additionally, the shelf life of ripened cheese is influenced by salt content. According to the results in Figure 1C, it was observed that there were significant differences in salt content during the ripening period among all cheese treatments. The control sample of cheese had the highest salt content compared with all other treatments until the end of the ripening period. Furthermore, salt content increased as the ripening period progressed in all treatments. The results in Figure 1D show the relationship between salt percentage and cheese ripening period using simple linear regression (SLR). There was a high positive correlation between cheese salt percentage and ripening period progress; this trend was observed in all cheese experiments: C; y = 0.03x + 1.63.

### 3.3. Ash Content of Ras Cheese

There were significant differences (*p* < 0.05) in ash content between all treatments during the ripening period (Figure 1E). The control sample of cheese had the highest ash content compared with other treatments, not only when fresh but also during the ripening period. Additionally, results illustrate the progress of the ripening period by the increase in ash content of cheese treatments. To better understand the relationship between ash content and ripening period, the simple leaner regression (SLP) was used (Figure 1F). The results showed that there was a high correlation between ash content and the progression of the ripening period in all Ras cheese treatments, while C, the control sample of cheese, had the highest positive correlation as evidenced by the equipment C; y = 0.41x + 6.06.

### 3.4. The Fat Content of Ras Cheese

There were significant differences in fat content between one-month-old treatments (Figure 2A). However, there are no significant differences between treatments at all time points, except during the first month of the ripening period. During the first month of the ripening period, T2 had a low fat content, whereas T4 had the highest fat content. The fat percentage in all cheese treatments increased as the ripening period progressed. There were no significant differences in the cheese at the end of the storage period. To illustrate the relationship between fat percentage and ripening period, simple leaner regression (SLR) was used (Figure 2B). It demonstrated that fat percentage and the ripening period had a positive correlation; this trend was much more pronounced in C as well as T4 C cheese, which had a high positive correlation. However, there was a low positive correlation. [T4; −0.09x + 3.49]. Furthermore, results showed that T1 and T2 cheese did not correlate with the ripening period.

### 3.5. Total Nitrogen Content of Ras Cheese

The results showed that there were significant (*p* < 0.05) differences in the total nitrogen percent while fresh and throughout the ripening period among all treatments (Figure 2C). T3 had the highest total nitrogen percentage throughout ripening compared with others. It could be observed that after 6 months of ripening, T3, as well as T4 cheese, had the highest total nitrogen percentage, while T1 cheese had the lowest. T4; y = 0.09x + 3.49. T1 and T2 cheese had a low positive correlation. Using a simple linear regression, the results shown in Figure 2D provided a better understanding of the relationship between total nitrogen percentage and ripening period. Positive regression was observed in all cheese treatments, but it was more pronounced in C, T3, and T4 cheese, which had a high positive correlation as shown in equation C; y = 0.11x + 3.18, T3; y = 0.07x + 3.76, and T4; y = 0.09x + 3.49, while T1, as well as T2, had a low positive correlation.

### 3.6. Acidity and pH Values of Ras Cheese

Throughout the ripening period, there were significant differences in acidity and pH values (*p* < 0.05) in all treatments (Figure 2E,G). There was a lower acidity development and a slight increase in pH values in all cheeses after one month compared with fresh samples. It was noticed that T2 cheese had the highest acidity at the beginning and during the ripening period. While control cheese had the lowest acidity compared with other treatments during the ripening period, it had a reverse approach in pH values.

The simple linear correlation (SLR), as shown in Figure 2F,H, was used to better understand the relationship between acidity percent and pH value with the ripening period. Both acidity percent and pH values had a positive correlation with the ripening period. The acidity increased throughout the ripening period for all treatments and vice versa for pH values, as illustrated by the equation T4; y = 0.073x + 5.76. As shown in equation C, the acidity tendency was more pronounced in the C treatment; y = 0.015x + 0.85.

### 3.7. Sensory Evaluation of Ras Cheese

The sensory results indicated that the sensory parameters of texture, color, and flavor, as well as the overall acceptability of cheese treatments, showed no significant differences (*p* < 0.05) throughout the ripening period (Figure 3A,C,E,G), although these parameters increased as the ripening period progressed. By prolonging the ripening period, the overall scores of cheese treatments C, the control samples of T1, T2, T3, and T4 cheese were 84.84, 84.75, 86, 87.17, and 88.25, respectively. Generally, all cheese samples were accepted by judges. Results illustrated there was a positive correlation between the sensory properties of Ras cheese treatments and the ripening period (Figure 2D,F,H and Figure 3B). Control Ras cheese had a high positive correlation in all sensory properties as shown in the equations [texture C; y = 0.13x + 2.15, color C; y = 0.14x + 2.60, and flavor C; y = 0.17x + 2.32] except in overall acceptability, which had week positive correlation C; y = 1.19x + 77.95. Results also revealed the flavor of all treatments had a high positive correlation with the progress of the ripening period that was more observed in the T2 treatment as shown by the equation T2; y = 0.20x + 2.21.

### 3.8. Volatile Compounds of Ras Cheese

Volatile compounds of the Ras cheese samples, which have the main effect on the cheese flavor, were identified. In the headspace of extracts, 38 volatile compounds were identified and classified into chemical groups such as ketones, esters, aldehydes, alcohols, and acids. The results showed that at the exact retention time, different volatile compounds had corresponding concentrations expressed as a percentage of the peak area of the total separations. Volatile compounds were identified using purge-and-trap TDCT GC-MS. Many different compounds were detected and characterized in the cheese samples.

The data in Figure 4 show the volatile compounds. The control sample of Ras cheese contains 1.0170% esters, 0.2169% ketones, 0.1441% alcohols, 0.2389% aldehydes, 41.1161% fatty acids, and 57.2669% other compounds. Meanwhile, T1 Ras cheese contains 0.7818% esters, 1.6405% ketones, 9.2227% alcohols, 0.00% aldehydes, 23.0126% fatty acids, and 65.3424% other compounds. T2 Ras cheese contains 16.1002% esters, 0.00% ketones, 0.00% alcohols, 0.00% aldehydes, 64.1896% fatty acids, and 19.7102% other compounds. T3 Ras cheese had 12.7301% esters, 1.8336% ketones, 3.5831% alcohols, 1.2085% aldehydes, 78.5561% fatty acids, and 2.0887% other compounds. Moreover, T4 had 9.3850% esters, 2.4810% ketones, 5.5905% alcohols, 0.9602% aldehydes, 66.2153% fatty acids, and 15.3680% other compounds.

Figure 5 shows the percentage of volatile compounds in treatments. It could indicate that T3 cheese had a greater percent of (2-pentanone,4-hydroxy-4-methyl, Oliecacid, eicosyl ester, Nonadecanoic acid, 2-Hexadecanol, Benzaldehyde, 3-benzyloxy-2-fluoro-4-methoxy, Myristic acid, n-Hexadecanoic acid, Oleic Acid, Hexadecanoic acid, 2-(octadecyloxy) ethyl ester, Triarachine, and Dodecyl cis-9,10-epoxyoctadecanoate. While T2 cheese had higher percent of n-Hexadecanoic acid, Oleic Acid, Z-8-Methyl-9-tetradecenoic acid, Z-8-Methyl-9-tetradecenoic-1-olacetate, l-(+)-Ascorbic acid 2,6-dihexadecanoate, Octadecanoic acid, 2,3-bis[(1-oxotetradecyl) oxy] propyl ester, Octacosane, Heptacosane1, Heptacosane 2 and Hexadecanoic acid, 2-hydroxy-1-(hydroxymethyl) and ethyl ester. Likewise, T4 had a higher percent of n-Hexadecanoic acid, p-Xylene, 2-Pentadecanone, trans-13-Octadecenoic acid, Tetratriacontane, Hexadecane, 1,1-bis(dodecyloxy), and tert-Hexadecanethiol. However, it had lowest percent of Oleic acid as well as 4-Allyl-2-t-butyl-4-methyl-1,3-oxathiolan-5-one. Data also indicated that T1 cheese had a greater percent of 4-Allyl-2-t-butyl-4-methyl-1,3-oxathiolan-5-one, Tetradecanoic acid, cholesterol, Estra-1,3,5(10)-trien-17a-ol, and terat-Hexadecanethiol, and furthermore that it had a modest percent of Myristic acid, n-Hexadecanoic acid, and z-8-Methyl-9-teteradecenoic-1-olacetate. On the contrary, some volatile compounds were not detected in T3 cheese while C cheese had a higher percent of it, such as p-Xylene, Hexadecanal, 2-methyl-, Glycerol 2-acetate 1,3-dipalmitate, 10-Undecenoic acid, octyl ester, Octadecanoic acid, 2-hydroxy-1,3-propanediyl ester, Palmitic acid, Oxirane, tetradecyl- and Hexadecanoic acid, and 2-hydroxy-1-(hydroxymethyl)ethyl ester).

Principle Component Analysis (PCA) was applied to the volatile compounds data and the similarity map was defined by principal components which described the total variance in the data as shown in Figure 6A,B. It could be observed that cheese treatments are classified into 4 groups. All cheese treatments were of a closely similar cluster in each region of the PCA, and the cheeses varied pronouncedly. T1 and T4 Ras cheese are in the same region of the PCA, as they contain the following compounds (Hexadecane, 1,1-bis dodecyloxy-Tetradecanone-Tetradecanoic acid- 4-Allyl-2-t-butyl-4-methyl-1,3-oxathiolan-5-one-Estra-1,3,5(10)-trien-17á-ol-tert-Hexadecanethiol-cholesterol). Meanwhile, C cheese contains the following compounds (Hexadecanoic acid, 1-(2-aminoethoxy) ethanediyl- ester-Octadecanoic acid, 2-hydroxy-1,3-propanediyl ester-10-Undecenoic acid, octyl ester-Palmitic acid-trans-13-Octadecenoic acid-p-Xylene-2-Pentadecanone-Hexadecanal, 2-methyl-Glycerol 2-acetate 1,3-dipalmitate-oirane, tetradecyl). T2 Ras cheese contains the following compounds (n-Hexadecenoic acid-Myristic acid-Z-8-Methyl-9-tetradecenoic acid-l-(+)-Ascorbic acid 2,6-dihexadecanoate–Heptadecane–Octacosane-Hexadecanoic acid, 2-hydroxy-1-(hydroxymethyl)ethyl ester-Octadecanoic acid, 2,3-bis [(1-oxotetradecyl)oxy] propyl ester). Meanwhile, T3 Ras cheese contains the following compounds (2-Hexadecanol-Benzaldehyde, 3-benzyloxy-2-fluoro-4-methoxy-Nonadecanone-2-Pentanone, 4-hydroxy-4-methyl-Dodecyl cis-9,10-epoxyoctadecanoate-Oleic Acid-Hexadecanoic acid, 2-(octadecyloxy)ethyl ester-Dodecanoic acid–Triarachine-Oleic Acid, ethyl ester).

## 4. Discussion

Ras cheese is one of the most traditional Egyptian hard cheeses. The quality of Ras cheese is determined by its physio-chemical characteristics and sensory properties that are attributed to its consumer acceptability. The steady properties are affected by compounds that are released by chemical reactions [13]. Additionally, the characteristics of the flavor of matured cheese are influenced by the degradation of casein and lipolysis [34]. Furthermore, the degradation that occurs in amino acids due to the starter culture pathways throughout the ripening period affects the flavor of matured hard cheese [35].

Raising the intake of sodium in food has been claimed to be a major contributor to the increase in the incidence of hypertension and cardiovascular diseases [36]. It has been recommended that the risk of hypertension be minimized. The daily intake of salt should be reduced in the diet by using different techniques in salting Ras cheese to reduce salt intake without influencing its characteristics or its score of acceptability relative to all cheese. This was more pronounced in treatment T3 cheese that was salted in the brine solution for 24 h, which possessed good characteristics, followed by T4 cheese that was salted in the brine solution for 12 h followed by dry salting.

Changes in moisture content in all Ras cheese treatments throughout the ripening period could be due to the evaporation of water from its surface [3,37]. However, the decrease in pH values and increase in acidity may be due to the metabolic activity of lactic acid bacteria [33]. Cheese is a biochemically energetic product that changes significantly during its ripening period. The production of flavor compounds that occur during the ripening period of cheese has different characteristics for each variety of cheese. These compounds are affected by the degradation of cheese contents that happened via a complex process involving milk’s enzymes, starter cultures, and rennet, as well as secondary microflora [38,39].

The changes in sensory properties were increased and gradually enhanced throughout the ripening period. All cheese treatments had higher scores at the end of the ripening period, which might be due to partial proteolysis of the cheese, as well as enhancement of flavor due to the balance between components released by enzymic reactions that occur during the ripening period. These results are in agreement with Abou Donia 2002 and Ayad et al. 2004 [1,32].The positive values of the violate compounds refer to the production of different compounds as a result of the metabolic activities of the starter culture, while the negative values indicated that the production of higher quantities in the control than in the experimental extracts might be due to the degradation of the inoculated starter culture [40].

A synchronized and steady ripening process compels a sequence of microbiological, biochemical, and chemical reactions, leading to products with desirable flavor. The flavor-forming abilities depend on the enzymes present, so different flavors could be obtained [41]. The differences in compounds between treatments and control were assessed. The increase in compounds refers to the metabolic starter culture that produced a net of compounds during its activity [42]. Alcohols and aldehydes identified in the cheese were a result of the catabolism of amino acids [43]. While different concentrations of the ketones varied between the different cheese samples, methyl-ketones were formed via enzymic oxidative decarboxylation of fatty acids (FA) [44].

There were four different alcohols that were present in the cheese treatments. These compounds were created by various metabolic routes such as the metabolism of lactose and amino acid degeneration of methyl ketones, and linoleic as well as linolenic acids [45]. The important volatile compounds that perhaps contribute to the flavor of cheese are alcohols. From the quantitative side, alcohols are considered the most abundant chemical group in some kinds of cheese. The concentration of alcohol in cheese fluctuates during ripening [46]. Differences in percentage of ethyl-esters were also detected between cheese treatments. These are due to enzyme analysis or chemical interactions between FA and primary alcohols. However, esters noticed in cheese, are considered important compounds in the flavor of the cheese [47,48].

Throughout the cheese, ripening proteolysis is considered the main biochemical reaction that affects the flavor compounds of cheese. Proteolysis is due to releasing amino acids that are subsequently catabolized to different volatile flavor compounds [49]. Catalyzation of enzymes from the coagulant milk and the starter culture’s enzymes are considered the main sources of proteolysis in cheese [17]. Caseins are hydrolyzed mainly by the coagulant into large and intermediate peptides that are degraded by the coagulant and enzymes from LAB which has a greater role in protein breakdown [50].

The optimal growth of *Lactococci* in milk mainly depends on their proteolytic systems, while milk has limited concentrations of free AA and peptides, so high peptidase expression provides the proteolytic activity of *Lactococcus lactis* in the high protein of milk. The breakdown of peptides indicates a positive contribution to flavor development. The formation of small peptides in cheese may be due to hydrolyzing the large peptides that are synthetic via chymosin or plasmin. Furthermore, the resulting peptides are degraded to amino acids by a pool of cytoplasmic peptides [51].

Amino acids are one of the important sources of flavor production by LAB that could imitate catabolism by transamination amino group acceptor as α-keto acid that is required for amino-transferases [52,53]. Lactobacilli convert amino acids to α-keto and hydroxy acids that could be converted to carboxylic acids via *Lactococcus*. Similarly, [54] revealed the production of high levels of branched-chain aldehydes, which are responsible for the development of flavor by cooperative completion of a metabolic pathway between two strains of *L. lactis* in milk. *Lactococcus* starters were combined with peptidases to increase the peptidolytic activity throughout cheese ripening, thus accelerating and diversifying proteolysis in cheese.

**Figure 7:**
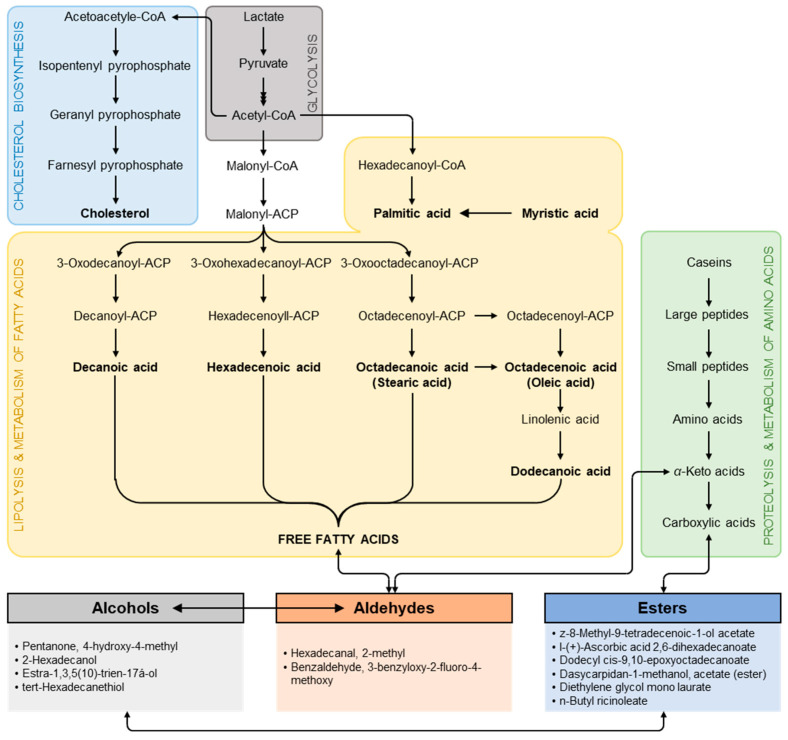
Schematic of the impact of salting techniques on the volatile organic compounds and other compounds of Ras cheese. ACP: acyl-carrier protein.

The flavor of the cheese is influenced by the release of free fatty acids (FFA) throughout ripening due to the lipolytic breakdown of the triglycerides of milk fat [55] as represented in Figure 7 which illustrates the degradation of Ras cheese compounds during the ripening period that also affected with salting technique. Additionally, intracellular enzymes could release from the starter bacteria in cheese, caused by autolysis lipolytic during ripening. The flavors of cheese made from pasteurized milk and lactic acid bacteria (LAB) culture would contribute more to lipolysis than endogenous enzymes. Likewise, mono and di-glycerides produced by the hydrolysis of triglycerides could be hydrolyzed by some esterases. Esterases carry out the hydrolysis and synthesis of ester bonds and can be divided into lipases and non-lipolytic esterases [56].

Lactic Acid Bacteria LAB culture can possess esterases and lipolytic activity that are mainly due to intracellular enzymes capable of hydrolyzing fat in milk and cheese. Despite the weak lipolytic system of *Lactococci* and *Lactobacilli* and their enzymes, they could be responsible for the liberation of significant levels of free fatty acids (FFA) in cheese because of the manufacturing of cheese without the addition of the starter culture or with low fat, which leads to lower levels of FFA [57].

Esters are considered one of the abundant compounds in cheese that are produced via the interaction between FFA and alcohols. Although methyl, propyl, and butyl esters have been in existence in cheese, the most common alcohol available for this interaction is ethanol, and therefore ethyl esters are the prominent esters in cheese. Esters are one of the compounds that are responsible for flavors in cheese, which are formed during direct esterification of alcohols and carboxylic acids or by alcoholysis [20]. Therefore, the composition of the milk and reaction conditions, such as pH degree, which is affected by the content of salt, might be an important factor affecting the ester compounds’ formation. Additionally, aldehydes originate from the degradation of amino acids, while ketone compounds are formed by enzymic oxidation of FFA to *β*-ketoacids, followed by decarboxylation to ketones [58,59].

Salting is an important process in the manufacturing of all kinds of cheeses. Salt has a vital role in cheese since it is considered a preservative substance in cheese as well as an important factor that affects the quality of cheese. The addition of salt to cheese dominates the growth of lactic acid bacteria as well as desisting offensive microbial growth that may lead to the appearance of off-flavor [60]. So, salt does not act as an antimicrobial agent directly. The concentration of salt added to cheese affects the growth rate of both lactic acid bacteria and non-starter lactic acid bacteria (NSLAB). The technique of dry-salted cheese, as in T1, T2, and the control sample of cheese, caused a rise in acidity and lessening of pH value, hence lactose residual in the cheese matrix that was utilized via non-starter lactic acid bacteria at the early period of ripening [8].

Furthermore, salt has a role in the hydration of casein since the low concentration of salt leads to an increase in casein hydration. Additionally, salt affects the water-holding capacity of the casein curd [59]. Salt at higher concentrations, as appeared in some experiential treatments, would decrease the casein hydration, likewise in control cheese, and consequently harden the cheese [8]. The concentration of salt affects the activity of the proteolytic enzymes in cheese. Additionally, the water activity, which in turn reduces the activity of the enzyme, could lessen to cause an increase in salt concentration in cheese, which affects the formation of volatile compounds in cheese.

While in T3 and T4 cheese, which were salted by brine solution, the net migration of Na^+^ and Cl^−^ ions that would be from the brine solution inside the cheese matrix could happen as an effect of the osmotic pressure that occurs because of the difference in concentration of NaCl in the cheese moisture and that in the solution. Therefore, the movement of moisture would be from the center of the cheese to the surface, and tend to cause differences in other compounds which affect the number of volatile compounds that are formed, such as peptides, free amino acids, and free fatty acids [60]

On the other contrary, ions of Na^+^ and Cl^−^ would move from the surface toward the center of the cheese, and that would cause a retardation of the enlargement and the migration of salt and water movements through the cheese. This immigration is hindered by globules of fat as well as clusters of protein that cause the movement of ions, through complex means, to drift from one region to another. The dry salt technique could slightly dissolve the surface moisture, and pieces of the matrix could cause a contrary stream of whey moving from the center to the surface, which may be associated with the dissolution of the remaining crystals. Volatile compound rates in cheese samples are affected by different factors such as the type of cheese, the kind of milk, the weak methods of standard products, and ripening period. Furthermore, sensory perception is affected by different factors, such as the ratio of flavor contents [61].

## 5. Conclusions

The current study aims to investigate the effect of different salting techniques on the characteristics of Ras cheese and to reduce salt consumption, which is linked to a variety of health problems. It could be observed that a new way of salting did not cause any negative characteristics or off-flavor compounds. Our findings showed that T3 Ras cheese salting in a brine solution (24%) for 24 h, followed by T4 Ras cheese salting in brine solution 24% for 12 h, had better characteristics in the physio-chemical and sensory properties, as well as volatile compounds. This salting technique could be used with other types of cheese, such as semi-hard cheese. Moreover, it might be preferable for people who are on a diet or have health problems correlated with high levels of salt in food.

## Data Availability

Data is contained within the article.
